# Balancing immune responses: regulatory cells in eosinophilic gastrointestinal disorders

**DOI:** 10.3389/fimmu.2024.1372009

**Published:** 2024-07-29

**Authors:** Nassim Kheshtchin, Zahra Kanannejad, Zahra Ghahramani, Hossein Esmaeilzadeh, Najmeh Sepahi

**Affiliations:** ^1^ Department of Immunology, School of Medicine, Shiraz University of Medical Sciences, Shiraz, Iran; ^2^ Allergy Research Center, Shiraz University of Medical Sciences, Shiraz, Iran; ^3^ Hematology Research Center, Shiraz University of Medical Sciences, Shiraz, Iran; ^4^ Department of Pediatrics, School of Medicine, Shiraz University of Medical Sciences, Shiraz, Iran

**Keywords:** eosinophilic gastrointestinal disorders, inflammation, immune system, regulatory eosinophil, regulatory T-cells

## Abstract

Eosinophilic gastrointestinal disorders (EGIDs) are a group of conditions characterized by an abnormal accumulation of eosinophils in the gastrointestinal tract, leading to inflammation and tissue damage. Regulatory cells are a subset of immune cells that are crucial in maintaining the balance of the immune system and preventing the occurrence of autoimmune diseases. In EGIDs, regulatory cells are believed to play a key role in controlling the immune response and overseeing the growth and activation of eosinophils in the gastrointestinal tract. There is evidence indicating that regulatory T cells (Tregs) and regulatory eosinophils may play a role in suppressing the inflammatory response in EGIDs. Regulatory eosinophils are a subgroup of eosinophils that possess an anti-inflammatory role. Recent studies have shown that enhancing the number or effectiveness of regulatory eosinophils can reduce the severity of EGIDs. Regulatory eosinophils dampen inflammation through their regulatory mediators, such as galectin-10 and growth factor beta (TGF-β), which promote Treg expansion and inhibit effector T cell function. Further research on regulatory cells in EGIDs may have significant implications for the advancement of novel therapies for these uncommon and intricate disorders. The aim of this review is to provide complete view of the immune responses connected to EGIDs, examine the regulatory cells that control these responses, and evaluate their potential as therapeutic targets for EGID treatment.

## 1 Introduction

Eosinophilic gastrointestinal disorders (EGIDs), which include eosinophilic esophagitis (EoE), eosinophilic gastritis (EoG), eosinophilic enteritis (EoN), and eosinophilic colitis (EoC), are long term inflammatory conditions characterized by a large influx of eosinophils (1). EGIDs affect the gastrointestinal tract without any known causes for eosinophilia (e.g. drug reactions, parasitic infections, and malignancy). Patients with EGIDs may encounter various issues, such as abdominal pain, growth failure, gastric dysmotility, irritability, diarrhea, and vomiting. EGIDs are known to arise from a complex interaction of genetic and environmental factors (2).

Understanding the complex immune mechanisms that are responsible for driving EGIDs is crucial in order to develop precise therapeutic interventions. Over the past decades, significant advancements have been made in understanding the intricate immune reactions that are responsible for EGIDs (3–6). It is now widely recognized that chronic allergic reactions, primarily caused by food allergens, play a crucial role in the development of these disorders. The gastrointestinal microenvironment serves as a “site of conflict”. This microenvironment is characterized by the interplay between the gut microbiota, immune cells, and various other components such as epithelial cells, stromal cells, and extracellular matrix (7). The gut microbiome, consisting of trillions of microorganisms, has a profound impact on the development and function of the immune system. Specific gut bacteria and their metabolites can modulate the activity and differentiation of immune cells, including T cells, B cells, natural killer (NK) cells, and innate lymphoid cells (ILCs) (8). Nevertheless, in the midst of this inflammatory environment, a compensatory factor arises in the form of regulatory cells. Regulatory T cells (Tregs) and other immune regulatory cell populations play a critical role in modulating immune responses and preventing excessive tissue damage (9). Tregs, identified by the presence of forkhead box P3 (FoxP3) and CD25, inhibit immune activation through multiple mechanisms, such as the release of anti-inflammatory cytokines and direct cellular interaction. In addition, regulatory eosinophils and regulatory myeloid cells play an active anti-inflammatory role in immune regulation within the gastrointestinal tissues (10–12).

This article aims to provide a comprehensive overview of the immune mechanisms involved in EGIDs, with a specific focus on the regulatory cells that are vital for preserving immune balance. Comprehending the distinct clinical presentations and diagnostic characteristics for each subtype is essential for the precise recognition and treatment of these disorders.

## 2 EGIDs classification

Dr. R. Kaisjer first documented eosinophilic gastroenteritis in 1937 while analyzing surgical specimens obtained from various regions of the gastrointestinal tract (13). In the 1990s, several case series documented instances of dysphagia in adults and refractory reflux symptoms in children. The presence of eosinophilic infiltration was the distinguishing factor between these cases and gastroesophageal reflux disease (GERD). As a result, EoE was identified as a distinct condition in both adult and pediatric patients (14). Primary EGIDs are a diverse set of conditions marked by inflammation induced by eosinophils in various parts of the gastrointestinal tract, such as the esophagus, stomach, duodenum, ileum, large intestine, and bile ducts/pancreas. These disorders occur without any identifiable reason for the presence of eosinophils (1). EGIDs are categorized into distinct groups according to the degree of eosinophilic infiltration (15). Different types of EGID have varied histopathologic features, clinical signs, and eosinophil density.

### 2.1 Eosinophilic esophagitis

The development of EoE is influenced by a complex interplay of genetic, environmental, and immune system factors (16). The main reason for this condition is a problem with the esophageal epithelium function, where the interaction between food allergens, changes in the protective layer of the esophagus, and potential alterations in the esophagus microorganisms collectively enable the entry of food allergens through the protective layer. Subsequently, this process triggers the activation of receptors and inflammatory cells, including eosinophils. Immune system responses, specifically IgE-mediated and T helper (Th)2 responses, drive the dysregulation of key genes involved in epithelial barrier function and proliferation, including kallikreins (KLKs), calpain-14 (CAPN14), and anoctamin-1 (ANO1). Additionally, the interleukin (IL)-13/CCL26/eosinophil axis plays a crucial role in this dysregulation (17). Researchers have found that allergens, cytokines, microRNAs (miRNAs), chemokines, and polarization of Th2 immunity contribute to this dysregulation. A characteristic feature of EoE is the remodeling of the esophagus, which leads to impaired esophageal function and the potential for food bolus impaction (14). The symptoms closely resemble those of GERDs, such as heartburn, acid regurgitation, epigastric or chest pain, dysphagia, food impaction, vomiting, and weight loss. The symptoms may differ based on the age of the affected individual (14). In young children, EoE may present more with non-specific gastrointestinal symptoms like vomiting, abdominal pain, and feeding difficulties. As children get older, the presentation tends to shift more towards dysphagia and food impaction, which are more common in adolescents and adults. This change in presentation is thought to be related to the maturation of the esophageal structure and function over time.

The endoscopic characteristics may encompass edema, linearly aligned creases (furrowing), mucosal rings (also referred to as feline esophagus), exudates, whitish papules, polyps, and strictures. A diagnosis requires the presence of at least 15 eosinophils per high-power field (Eo/HPF) in the esophagus, which can be found in the distal, mid, or proximal regions (18–20). Endoscopy can also be macroscopically normal (21). The histopathological characteristics comprise eosinophilic inflammation, eosinophil abscess, eosinophil surface layer, basal zone hyperplasia, dilated intercellular spaces, dyskeratotic epithelial cells, and lamina propria fibrosis. Furthermore, immunostaining can detect markers such as monocyte chemoattractant protein (MCP), eosinophil cationic protein (ECP), IgE, and tryptase (22). Peripheral blood eosinophilia may or may not be detected as a laboratory measurement. Differential diagnoses for EoE include infection, hypereosinophilic syndrome (HES), neoplasm, connective tissue disease/systemic sclerosis, small vessel vasculitis, proton pump inhibitor-responsive esophageal eosinophilia, and drug reactions (23, 24). An elemental diet, step-down approaches using 6, 4, and 2 food elimination diets (FED), topical glucocorticoids, and proton pump inhibitors have all been used to treat EoE (25).

### 2.2 Eosinophilic gastritis

EoG is defined by the presence of eosinophilic inflammation in the stomach, which may also spread to the esophagus. The clinical manifestations of mucosal EoG encompass vomiting, abdominal pain, diarrhea, malabsorption, protein-losing enteropathy, iron-deficient anemia, failure to thrive, and melena. Gastric involvement is observed in 38% of instances, duodenal involvement in 62% of instances, and extension to the ileum in roughly 72% of instances (16). Patients with EoG often exhibit increased blood eosinophil counts. In addition to eosinophils, there is an accumulation of MIB-1^+^ and CD117^+^ mast cells, FoxP3^+^ Tregs, and activated T cells in EoG. Researchers have observed upregulated levels of Th2 cytokines (IL-4, IL-5, and IL-13), IL-17, IL-18, and the eosinophil-related chemokine eotaxin-3 in EoG. Patients with EoG who have allergies possess a group of IL-5^+^ Th2 cells that are specifically targeted towards food allergens. Exposure to food triggers and stimulates the development of IL-5^+^ Th2 cells, leading to inflammation in the gut in EoG (26, 27). Notably, EoG has a distinct transcriptome with minimal overlap with EoE (10).

In normal conditions, the average eosinophil density in the stomach’s lamina propria is 2Eo/HPF, with no intraepithelial eosinophils observed. In the lamina propria of the duodenum and ileum, the densities are 10/HPF and 13/HPF, respectively, with few eosinophils inside the epithelium. The diagnostic criteria for EoG include the presence of at least 30 Eo/HPF in at least 5 HPFs, or at least 70 Eo/HPF in at least 3 HPFs for the stomach. For the duodenum, the criteria is at least 52 Eo/HPF, and for the ileum, it is at least 56 Eo/HPF (28). Examination of tissue samples from patients with EoE may show the presence of eosinophilic inflammation in different layers, flattened villi, and positive immunostaining for markers such as MCP, ECP, IgE, and tryptase. The presence of peripheral blood eosinophilia is not universally observed in cases of EoG.

EoG is strongly linked to allergic disorders, and people who have it usually have a predominant allergic phenotype with high levels of IgE and Th2 cell involvement. The long-term progression of EoG remains poorly understood. Various therapeutic interventions are available for the treatment of EoG, including elemental diets and the administration of topical and systemic glucocorticoids such as fluticasone, prednisolone, and budesonide. Mast cell stabilizers, such as Cromolyn, and leukotriene receptor antagonists, like montelukast, are also used to treat EoG. Patients with established allergies to particular foods frequently utilize dietary limitations in addition to medication-based methods for treating EoG. Patients with EoE may limit typical allergens such as dairy, eggs, soy, wheat, nuts, seafood, and red meats.

### 2.3 Eosinophilic colitis

The colonic form accounts for approximately 88% of eosinophilic diseases (16). The underlying mechanism of EoC involves abnormal hypersensitivity, where infants may have an association with food allergens, while adults may have involvement of T lymphocytes. EoC is distinguished by clinical manifestations including abdominal pain, diarrhea, weight loss, loss of appetite, bloody diarrhea, constipation, mucosal lining damage, and impaired nutrient absorption (29). It is also characterized by the infiltration of eosinophils into the colon, accompanied by increased levels of IgE and IL-5 in the bloodstream. Peripheral blood eosinophilia may not always be present. Endoscopic examination may reveal erythema, loss of vascularity, and lymphonodular hyperplasia. The diagnostic criteria require the presence of at least 100 Eo/HPF in the cecum/ascending colon, at least 84 Eo/HPF in the transverse/descending colon, and at least 64 Eo/HPF in the sigma/rectum. In normal circumstances, the average density of eosinophils in the large bowel is 8-30 per HPF (30, 31).

The histopathological features of EoC include eosinophilic cryptitis or crypt abscesses, alterations in crypt structure, elevated levels of eosinophils within the epithelium, and the presence of eosinophils in the muscularis mucosa and submucosa. In addition, the lamina propria shows infiltration of eosinophils and lymphocytes, while eosinophils are also present within the crypts. It is crucial to distinguish EoC from various other conditions, including infection, HES, ulcerative colitis, Crohn’s disease, connective tissue disease/Sjögren’s syndrome, small vessel vasculitis, systemic mastocytosis, and drug reactions (32). It is associated with allergic disorders, particularly with a predominant allergic phenotype characterized by T-cell involvement. Elemental diets are one way to treat EoC because they provide complete nutrition. Other treatments include topical and systemic glucocorticoids, antihistamines, drugs that block leukotriene receptors, and drugs that target IL-5 and IgE levels (33, 34).

## 3 The main immunological features of EGIDs

**Figure 1:**
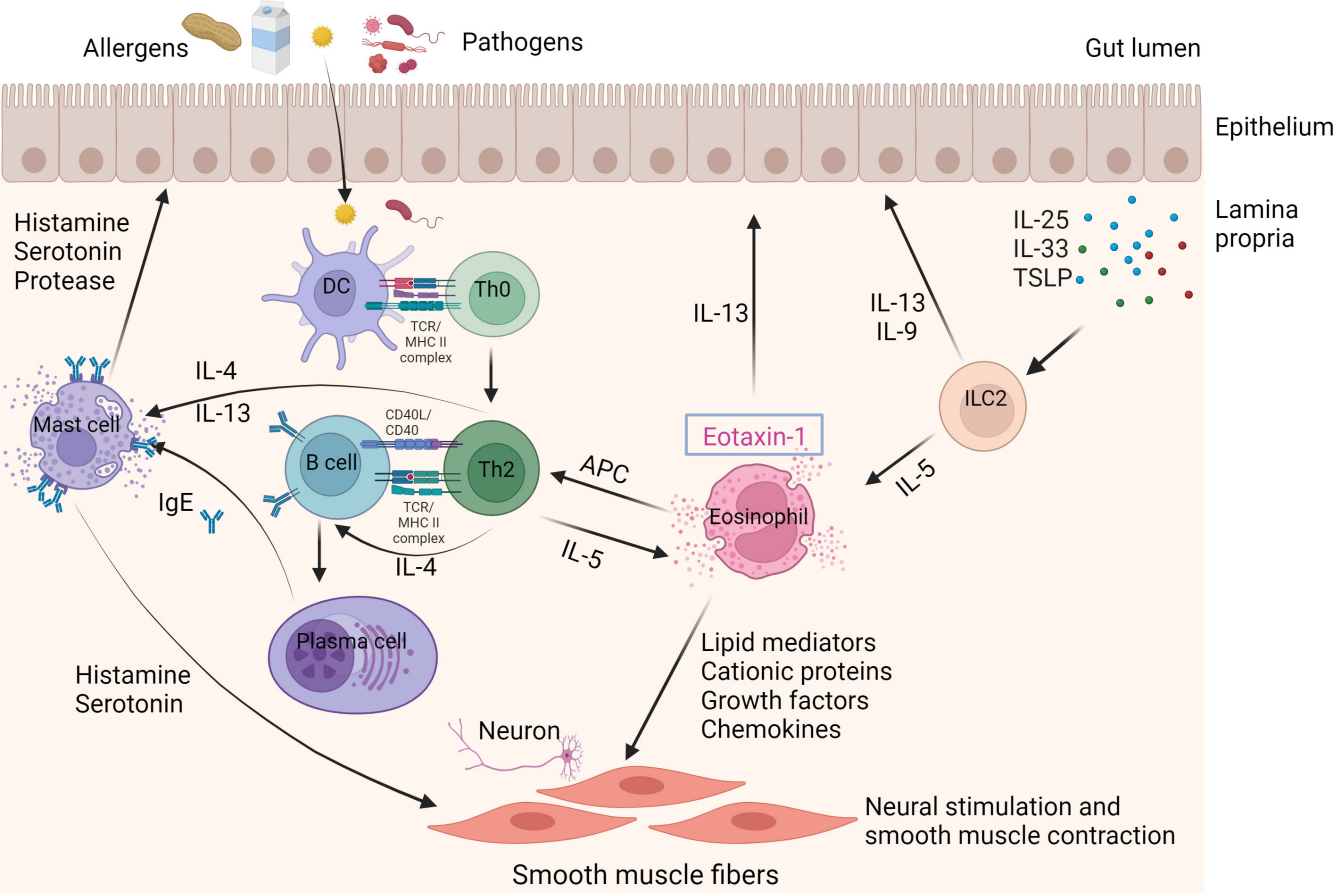
Immune mechanisms in EGIDs. The eosinophil is the primary contributor to EGIDs. Eosinophils are stimulated by allergens and/or pathogens that enter the epithelial barrier from the gut lumen. Furthermore, the activation and recruitment of ILC2 cells in response to stimulation by IL-23, IL-33, and TSLP can also trigger eosinophils. Eosinophils can serve as antigen-presenting cells (APCs) to Th2 lymphocytes. Th2 lymphocyte-derived cytokines, such as IL-4 and IL-13, stimulate the process of immunoglobulin class switching, which results in the production of proallergic IgE antibodies. Cytokines produced by Th2 cells, such as IL-5, stimulate eosinophils. The release of eosinophil granule proteins leads to the activation of mast cells and an increase in epithelial permeability. The release of IL-4 and IL-13 from Th2 cells, as well as IgE secreted by B cells, activates mast cells. Activated mast cells release histamine, serotonin, and protease, which are linked to eosinophil mediators. This leads to neural stimulation and contraction of smooth muscles, causing gastrointestinal symptoms like abdominal pain and bloating. APCs; ILC2, Innate lymphoid cells; TSLP, Thymic stromal lymphopoietin.

EGIDs characteristically exhibit an increased number of activated eosinophils in gastrointestinal tissues (35). The activated eosinophils in the gastrointestinal tract generate mediators that result in tissue damage and give rise to the symptoms of EGIDs (36). The precise etiology and pathogenesis of EGIDs have yet to be fully clarified. Nevertheless, the connection between EGIDs and food allergens suggests a complex interaction between genetic and environmental elements. The majority of patients with EGIDs exhibit a familial history of atopy or positive skin tests for different food allergens, which supports the concept of eosinophil accumulation in the digestive tract as a response to dietary or environmental antigens (37–39). Interestingly, an allergen-free diet might occasionally be considered a therapeutic strategy for EGIDs (40, 41). Allergic sensitization to food allergens is generally defined as a type 2 effector phase directed against food allergens, involving the expression of Th2-promoting mediators including thymic stromal lymphopoietin (TSLP), IL-25, and IL-33, activation of ILC2 and Th2 cells, and subsequent generation of allergen-specific IgE. The IgE is then released and binds to high-affinity IgE receptors (FcϵRI) on the outside of mast cells and basophils in tissues. This binding prompts these cells to discharge pre-existing histamine and other pro-inflammatory substances upon subsequent contact with a particular allergen. These substances are responsible for the swift onset of allergy symptoms. Subsequently, these substances generate further pro-inflammatory mediators such as leukotrienes, platelet activating factor (PAF), IL-4, IL-5, IL-13, tumor necrosis factor (TNF), and chemokines, resulting in a delayed inflammatory reaction characterized by the attraction of various immune cells, primarily eosinophils, basophils, and T cells (**Figure 1**) (42).

IL-5 is crucial for the growth, final differentiation, maturation, and survival of eosinophils (43). Research has also found that IL-5 is very important for the release of eosinophils from the bone marrow into the bloodstream and for their movement in the gut and lungs when they are exposed to allergens (44, 45). EGID patients have been reported to exhibit increased expression of Th2 cytokines (46). It has been demonstrated that T cells in patients with EoG differentiated to IL-4^-^IL-5^+^ Th2 cells, while the IL-4^+^IL-5^-^ subpopulations were dominant in allergic patients, which may explain, at least in part, the different pathophysiology of allergy and EGIDs (27). It is important to note that chemokines in the eotaxin family also mobilize eosinophils. Of these, eotaxin-1 is thought to be the most important chemotactic factor in the accumulation of eosinophils in lower eosinophilic gastrointestinal diseases, while eotaxin-3 is the main player in upper EGIDs (47, 48). Upon activation, eosinophils release a variety of pro-inflammatory substances, including cytotoxic granule proteins such as major basic protein (MBP), ECP, eosinophil peroxidase (EPO), and eosinophil-derived neurotoxin (EDN). They also release inflammatory cytokines like IL-1, IL-3, IL-4, IL-5, IL-6, IL-8, eotaxin, GM-CSF, TNF, and leukotrienes, which promote inflammation and tissue damage (49–52). However, as indicated earlier, only a part of EGID patients experience clinical and histological remission through dietary modifications. This observation implies that factors other than allergens, such as non-specific tissue damage and parasitic and bacterial infections, may be responsible for initiating type 2 responses and activating eosinophils in EGIDs (53). Moreover, patients without a history of atopy might present with autoimmunity, which suggests that different immune pathways or even autoimmune pathways drive eosinophilia (54). Gastrointestinal dysbiosis, infections, and anatomical malformations are among other possible causes of EGIDs (35, 55). Defective immunoregulatory mechanisms can also trigger the development of EGIDs, similar to other inflammatory disorders.

## 4 Anti-inflammatory activity in EGIDs

The immune system is essential for preserving the body’s overall well-being by protecting it from detrimental pathogens and inhibiting the onset of diseases. Nevertheless, in order to preserve a fragile equilibrium, the immune system also utilizes diverse regulatory mechanisms to prevent excessive immune reactions that may result in autoimmune disorders or persistent inflammation. Understanding immune regulatory mechanisms in both health and disease is critical for developing therapeutic interventions. Understanding the cellular components of immune regulatory mechanisms provides insights into the complex interactions and networks that govern immune regulation. Manipulating these cells or their functions holds promise for developing targeted therapies to restore immune balance and treat immune-related disorders such as autoimmune diseases, allergies, and chronic inflammation. Treg, dendritic cells, macrophages, and B cells are key cellular components involved in immune regulatory mechanisms. These cells have specialized functions in modulating immune responses, maintaining immune tolerance, and preventing excessive inflammation. Immunoregulatory cytokines such as IL-10, transforming growth factor beta (TGF-β), IL-4, IL-35, and IL-27 are a specific group of cytokines that are important for regulating immune responses and keeping the immune system balanced.

### 4.1 Regulatory T cells

FoxP3^+^CD25^+^CD4^+^ Treg cells are specific subsets of T cells that play a crucial role in preserving peripheral tolerance, preventing autoimmunity, and restricting chronic inflammatory diseases (9). Regulatory T cells possess various mechanisms to exert their inhibitory effects. These can be categorized into four fundamental modes of action: inhibition through inhibitory cytokines, inhibition through cytolysis, inhibition through metabolic disruption, and inhibition through modulation of dendritic-cell (DC) maturation or function.

EGIDs are chronic diseases characterized by the presence of inflammation that is rich in eosinophils. Th2 cells primarily control immune responses that trigger this inflammation, which is caused by allergens found in food and the environment. Multiple studies demonstrate that Treg cells play a crucial role in controlling immune responses induced by Th2 cytokines (56). There is limited data regarding the involvement of Tregs in the disease pathology of EGIDs, with most studies primarily examining EoE (57) (**Table 1**).

**Table 1:** Regulatory T cells in eosinophilic gastrointestinal diseases.

Disease	Subjects	Results	Reference
EoE	EoE (n=33) GERDs (n=7) HC (n=8)	↑FoxP3 expression in the EoE group ↑ Treg amount in esophageal tissue of EoE	(58)
EoE	EoE (n=10) GERDs (n=10) HC (n=10)	↑ Treg in EoE	(59)
EoE	EoE (n=10) GERDs (n=8) HC (n=10)	↑ The numbers of FoxP3^+^, CD25^+^, and CD8^+^ cells in EoE and GERD ↑ Eosinophil degranulation and micro-abscesses in EoE	(60)
EGE	EGE HC	↑ Treg in EGE ↑IL4, IL5, IL13, IL17, CCL26	(10)
EGIDs GERD	EGIDs (n=34) GERD (n=23) HC (n=25)	↑Serum level of TGF-β and IL-10	(12)
EoE	EoE (n=10) GERD (n=10) HC (n=8)	↑Treg numbers in EoE compared with GERD and control groups ↑ T lymphocyte in EoE	(57)

EoE, Eosinophilic esophagitis; EGIDs, Eosinophilic gastrointestinal diseases; HC, Healthy control; FoxP3, Forkhead box P3; Treg, T regulatory; GERD, Gastroesophageal reflux disease; EGE, Eosinophilic gastroenteritis; ↑, increased.

Most of these studies have documented an increase in the number of Treg cells, however a few studies have observed a decrease in Treg cell populations. Tantibhaedhyangkul et al. studied the immunoregulatory mechanisms involved in EoE in comparison to normal control (60). They found an increase in esophageal CD25^+^ FoxP3^+^ cells in children with EoE compared with normal controls as a part of a negative feedback mechanism responsible for regulating the inflammatory response triggered by external stimuli or allergen exposures. Other studies in this field have confirmed these results (59). The increased number of Treg cells in the esophageal tissue of individuals with EoE suggests that these Treg cells may be stimulated to inhibit eosinophilic activity. The persistence of the disease state in esophageal tissue raises the question of why it occurs despite the presence of Tregs. In an allergic airway disease model, the lung displayed infiltration of Tregs, resulting in the suppression of inflammation. However, despite the existence of these cells, airway hyperreactivity endured. Several studies have shown that Treg cells obtained from individuals with atopic conditions exhibit reduced suppressive activity in comparison to Treg cells from individuals without atopic conditions (58). Nguyen et al. showed that individuals with allergic asthma have attenuated chemokine signaling pathway in Treg cells (61). Therefore, the activation of eosinophils may induce the infiltration of Treg and T cells into the sites of inflammation in EGIDs, resembling allergic diseases. However, the inhibitory role of Treg cells may not be adequate to inhibit the development of the disease. In this regard, some studies have tried to assess Treg functionality along with Treg frequency in EGIDs. Fuentebella et al. conducted a study to assess IL-10 and TGF-β, which are essential for the suppressive function of Treg cells and are also known to be secreted by Treg cells (58). The study showed that IL-10 and TGF-β levels were lower in the esophageal tissue of people with EoE compared to a healthy control group. However, FoxP3 expression was higher in EoE patients. This implies that the Treg function may be compromised. It may be related to FoxP3 instability and impaired function in inflammatory conditions like EGIDs (62). Researchers have identified two separate categories of Tregs. Natural Tregs (nTregs) are considered a distinct sub lineage that emerges during thymus differentiation. On the other hand, T cell receptors (TCRs) stimulate the production of induced Tregs (iTregs) from naïve T lymphocytes in the presence of TGF-β, retinoic acid (RA), and IL-10. The gastrointestinal tract contains mostly induced iTregs, which are formed as a result of prolonged exposure to antigens like food or microbial antigens. In EGIDs, eosinophils in lamina propia have a crucial role in iTreg differentiation through the production of TGF-β and all trans’ retinoic acid (ATRA). Eosinophil-derived TGF-β was verified to promote Treg generation in ovalbumin-specific models. This function seems to specifically influence the peripherally induced gastrointestinal Treg population while having negligible impact on the thymus-derived Treg group (63). In inflammatory conditions, both nTregs and iTregs may undergo a loss of FoxP3 expression, a key marker of Treg identity, and differentiate into pro-inflammatory subsets, including Th1 or Th17 lymphocytes, thereby exacerbating inflammation (64, 65). Some FoxP3-negative Tregs may also differentiate into T follicular helper cells, which play a role in humoral immunity (66). The observed increase in Tregs with reduced functionality in the intestinal biopsies of patients with EGIDs could be attributed to the plasticity of Tregs and their conversion into T helper cells in the presence of inflammation.

In order to obtain a more comprehensive understanding of this phenomenon, it would be advantageous to analyze additional T cell subsets, such as Th1, Th2, and Th17, in tissue biopsies collected from patients with EGIDs. These investigations can yield valuable insights into the dynamics and interactions of different T cell populations in the context of EGIDs.

### 4.2 Regulatory eosinophils

**Figure 2:**
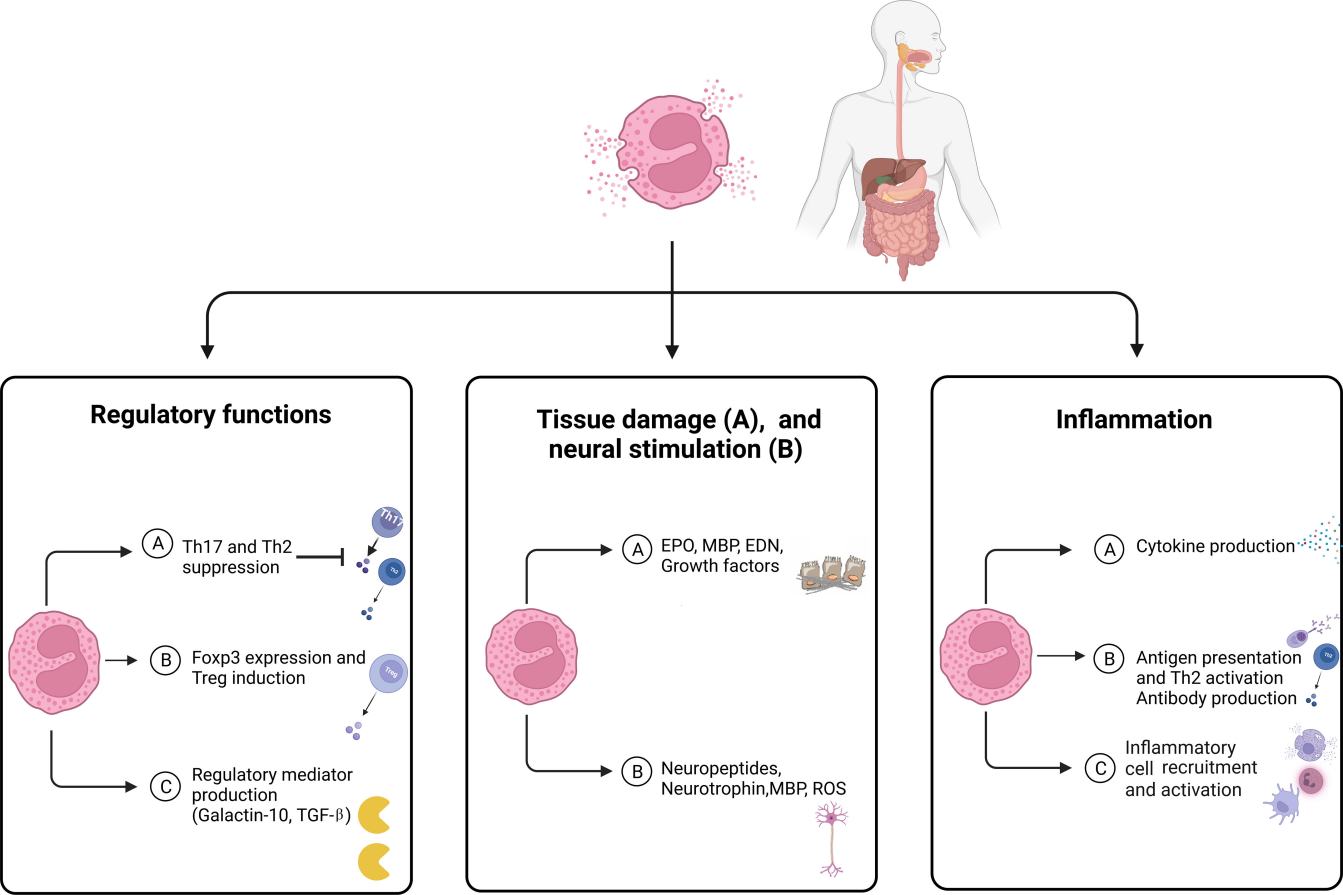
The main features of eosinophils in eosinophilic disorders. Eosinophils modulate tissue inflammation through the release of cytokines and lipid mediators, which enhance both adaptive and innate immune responses. Eosinophils serve as a reservoir of diverse proteins and cytokines that play a crucial role in the processes of fibrogenesis and angiogenesis, which contribute to tissue remodeling, repair, and fibrosis. Eosinophils have the ability to control the functioning and growth of nerve cells by releasing neuropeptides and neurotrophins, as well as MBP and ROS. Eosinophils play a role in regulating immune function and controlling inflammatory responses through the release of regulatory mediators and the expression of FoxP3. EDN, Eosinophil-derived neurotoxin; EPO, Eosinophil peroxidase; MBP, Major basic protein; ROS, Reactive oxygen species; TGF, Transforming growth factor.

Eosinophils are granulocytes capable of producing a diverse range of molecules stored in granules and lipid bodies throughout their cytoplasm. Eosinophils were historically perceived as cells capable of causing harm due to their remarkable ability to generate reactive oxygen species and secrete cytotoxic proteins (67). However, recent findings have revealed another function of eosinophils in the regulation of T-cell activity (**Figure 2**).

Studies by Roufosse and colleagues have shown that eosinophils can hinder the activation and growth of premalignant T cells linked to the lymphocytic variant of hyper-eosinophilic syndrome (68). This highlights the unrecognized role of eosinophils in modulating the activities of T cells. Studies on mice has found that eosinophils have a suppressive effect on the differentiation of Th17 cells in the small intestine and Th2 cells in Peyer’s patches (69).

Christine Lingblom and colleagues conducted studies to examine the presence of a regulatory subset of eosinophils. The research showed that eosinophils from healthy adults and people who have had hematopoietic stem cell transplants and developed chronic graft-versus-host disease (GVHD) can stop the growth of CD4^+^ and CD8^+^ T cells from a different donor by directly contacting these cells (70). In their study, Lingblom et al. identified a highly effective group of eosinophils in the blood of healthy adults known as the CD16^hi^ subset, which suppresses T cells in laboratory conditions (5). After co-culturing eosinophils with T cells stimulated by allogeneic leukocytes or CD3/CD28 cross-linking for 2 days, this particular eosinophil subset demonstrated elevated levels of CD16. Studies have shown that this subset represents 1-5% of all eosinophils and is more effective at suppressing T cell proliferation compared to the conventional CD16^neg^ eosinophils (71). The CD16^hi^ regulatory eosinophils also serve as the primary source of the immunoregulatory protein galectin-10, which acts as a suppressive molecule for T cells.

Research indicates that galectin-10 makes up around 7-10% of the total protein in eosinophils (72). Additionally, Galectin-10 has been discovered to confer Treg cells with the ability to suppress T cell activity (73). In a laboratory setting, Lingblom et al. observed a step-by-step process which eosinophils undergo when exposed to proliferating T cells. This process begins with the formation of immune synapses between eosinophils and T cells, which contain galectin-10. Next, cap-like accumulations of galectin-10 form on the eosinophil nuclear lobes. Finally, the eosinophils release galectin-10 along with nuclear DNA nets (69). Significantly, only the eosinophils that expressed CD16 released galectin-10 through these mechanisms. Nevertheless, the precise role of galactin-10 remains poorly understood.

In the context of EGIDs, there is limited information about the regulatory function of eosinophils. As with other studies on regulatory eosinophils, Lingblom and colleagues looked at how well eosinophils from EoE patients could suppress inflammation and how much FoxP3 they expressed (11). Eosinophils obtained from the blood of patients with EoE exhibited the ability to suppress T cell activity. Additionally, a portion of these eosinophils displayed the presence of FoxP3 in both the cytosol and nucleus. However, it was observed that these regulatory eosinophils in EoE had a lower capacity to suppress T cell activity compared to those found in healthy individuals. In this study, eosinophil lysates obtained from patients with EoE were used for immunoblot analysis. The analysis revealed the presence of two isoforms of human FoxP3 in eosinophils from EoE patients: the full-length isoform (FOXP3FL) and the isoform lacking exon 2 (FOXP3DE2). Both isoforms were found to exhibit suppressive activity in human Tregs and to work together in a coordinated manner.

Understanding the implications of the lower capacity of regulatory eosinophils in EoE is essential for developing targeted therapeutic strategies aimed at restoring proper immune regulation and reducing inflammation in the esophagus.

## 5 Conclusion

The immune system is a critical component in the development of EGIDs. Dysregulated immune responses involving eosinophils and Th2 cells, have been linked to the development and advancement of EGIDs through the action of their inflammatory mediators. However, a novel aspect of eosinophils has been discovered in recent years. Regulatory eosinophils, which possess immunosuppressive characteristics, have been recognized as a distinct subgroup of eosinophils. They play a role in modifying immune responses, working in conjunction with Treg cells. The existence of these regulatory cells in the gastrointestinal tract has the potential to stabilize immune responses and improve the symptoms of EGIDs. Therapeutic strategies focused on improving the function of regulatory eosinophils or increasing the number of regulatory T-cells show potential for reducing eosinophilic inflammation and reducing the severity of the disease.

## 6 Future directions

Despite significant advancements that have been made in understanding the function of regulatory cells in EGIDs, there are still several important areas that require additional research. Our understanding of the precise role of regulatory eosinophils in EGIDs is currently quite limited. Further research is required to investigate the precise molecules and signaling pathways that play a role in the regulatory function of eosinophils in EGIDs. Also, understanding how regulatory eosinophils interact with other immune cells will make it easier to find possible treatment targets for all eosinophilic diseases. In addition to these regulatory cells, other regulatory cells such as regulatory B cells and myeloid-derived suppressor cells may also help keep the immune system in check in EGIDs, just as they do in other allergic diseases. Nevertheless, there is a lack of existing data investigating their role in EGIDs. Investigating their participation and possible therapeutic uses could expand our understanding of immune regulation in EGIDs.

## Author contributions

NK: Investigation, Writing – original draft. ZK: Conceptualization, Investigation, Project administration, Supervision, Writing – original draft, Writing – review & editing. ZG: Investigation, Writing – original draft. HE: Writing – original draft. NS: Writing – review & editing.
